# The bidirectional link between HDL and COVID-19 infections

**DOI:** 10.1016/j.jlr.2021.100067

**Published:** 2021-03-17

**Authors:** Kenneth R. Feingold

**Affiliations:** Department of Medicine, University of California San Francisco, San Francisco, CA, USA

**Keywords:** SAR-CoV-2, COVID-19, high-density lipoprotein cholesterol, low-density lipoprotein cholesterol, apolipoproteins, infection, COVID-19, coronavirus disease 2019, HDL-C, high-density lipoprotein cholesterol, LDL-C, low-density lipoprotein cholesterol, SARS-CoV-2, severe acute respiratory syndrome coronavirus 2

## Effect of infections on lipid and lipoproteins

It is well recognized that gram positive and negative bacterial infections, tuberculosis, fungal infections, and parasitic infections result in changes in plasma lipid levels (1, 2, 3, 4, 5, 6, 7, 8, 9, 10, 11, 12). Of note, viral infections, such as HIV, Epstein-Barr virus, and Dengue fever, also similarly alter plasma lipid levels (13, 14, 15). Typically, infections decrease total cholesterol, low-density lipoprotein cholesterol (LDL-C), and high-density lipoprotein cholesterol (HDL-C) levels with either elevated triglyceride or inappropriately normal triglyceride levels for the decreased nutritional status that characteristically occurs with infections. As would be expected from the changes in lipid levels, apolipoprotein A-I, A-II, and B levels are also reduced (1, 7, 8). With recovery from infection, the alterations in plasma lipid levels return toward the baseline. The greater the severity of the infection, the greater the decrease in total cholesterol, LDL-C, and HDL-C levels (16, 17, 18). Numerous studies have shown that the degree of reduction in total cholesterol, HDL-C, and apolipoprotein A-I predict mortality in patients with severe sepsis (19, 20, 21, 22, 23, 24, 25).

Toward the end of 2019, a deadly new viral infection emerged caused by the severe acute respiratory syndrome coronavirus 2 (SARS-CoV-2), which resulted in coronavirus disease 2019 (COVID-19) (26). This virus rapidly spread throughout the world, leading to a worldwide pandemic. It is estimated that approximately 80% of COVID-19 infections are either asymptomatic or result in only mild symptoms, but in a significant percentage of patients, the infection leads to a severe respiratory illness requiring hospital care and respiratory support (26, 27). As of January 20, 2021, there have been over 2 million deaths worldwide according to the John Hopkins Coronavirus Resource Center. Older age, obesity, diabetes, cardiovascular disease, hypertension, and male gender are some of the factors that increase the risk of severe infection and death (26, 27).

As observed with other infections, numerous studies have reported a decrease in total cholesterol, LDL-C, and HDL-C levels and variable changes in triglycerides in patients with COVID-19 infections (28, 29, 30, 31, 32, 33, 34, 35, 36, 37, 38, 39). As expected, apolipoprotein A-I and B were also decreased (39, 40, 41). With recovery from COVID-19 infection, the lipid levels return toward levels present before infection (28, 29, 30, 42, 43). As expected, the greater the severity of the illness, the greater the reduction in LDL-C and/or HDL-C levels (29, 31, 32, 33, 34, 35, 36, 38, 42, 44, 45). LDL-C and HDL-C levels are inversely correlated with C-reactive protein levels, that is, the higher the CRP levels, the lower the LDL-C or HDL-C level (28, 29, 31, 45). Low LDL-C and/or HDL-C levels at admission to the hospital predict an increased risk of developing a severe disease. Increased mortality was observed in patients with low total cholesterol, LDL-C, and/or HDL-C levels at admission to the hospital, and in these very ill patients, lipid levels continued to decline during the hospitalization (28, 36, 38, 41, 43, 44, 45). A single study reported that the time to develop a negative RT-PCR test for SARs-CoV-2 was increased in patients with low HDL-C levels (46). Finally, HDL isolated from patients with COVID-19 infections displayed a blunted ability to protect against TNF-alpha–induced increases in endothelial cell permeability, vascular endothelial–cadherin disorganization, and apoptosis (39).

## Effect of lipid and lipoproteins on the risk for developing infections

A large number of observational studies have found that low total cholesterol, LDL-C, and/or HDL-C levels are associated with an increased risk of developing infections and sepsis (47, 48, 49, 50, 51, 52, 53, 54, 55, 56, 57, 58). For example, in a cohort of men (55,300) and women (65,271) in the Kaiser Permanente Medical Care Program who were followed up for 15 years, total cholesterol levels were inversely associated with infections requiring hospitalization or acquired in the hospital (52). It should be recognized that confounding factors could explain the association of low LDL-C and/or HDL-C with an increased risk of infection. Unrecognized disease, for example, pulmonary or gastrointestinal disorders, could decrease HDL-C and LDL-C levels and independently also increase the risk of infections and sepsis. In fact, in a recent study that found that low LDL-C levels were significantly associated with an increased risk of sepsis and admission to the ICU, the authors found that this association could be accounted for by comorbidities (60). Thus, more sophisticated studies, beyond observational studies, are necessary to demonstrate a causal relationship between low LDL-C and/or HDL-C levels with infections.

Several studies have taken a genetic approach, which reduces the risk of confounding variables, to determine if there is a causal relationship between lipoprotein levels and infections. Madsen et al using two common variants in the genes encoding hepatic lipase and cholesteryl ester transfer protein that regulate HDL-C levels found in 97,166 individuals from the Copenhagen General Population Study that low HDL-C increased the risk of infection (56). It was also noted in this study that high HDL-C levels were also associated with an increased risk of infection. Trinder et al using polygenic scores for LDL-C, HDL-C, and triglycerides in 407,558 individuals from the UK BioBank found that an increasing HDL-C polygenic score reduced the risk of hospitalizations for infections and sepsis induced mortality while LDL-C and triglyceride polygenic scores were not associated with the risk of hospitalization for infections or sepsis-induced mortality (61). This study did not find an increased risk of infection with high HDL-C levels. Finally, Walley and colleagues also reported that HMGCoA reductase and PCSK9 genetic variants that decrease LDL-C levels were not associated with an increase in mortality because of sepsis (59). Taken together, these studies suggest that low HDL-C levels may play a causal role in infections.

In the current issue of the *Journal of Lipid Research*, Hilser *et al*. (62) utilized the UK BioBank to examine the association of HDL-C measured between 2006 and 2010 and the development of COVID-19 infections in 2020. They compared hospitalized patients who tested positive for COVID-19 (n = 1,117) (ie, individuals with severe COVID-19 infections) with patients who tested negative for COVID-19 infections in either in-patient or out-patient hospital settings (n = 3,544). Results in the overall group were analyzed and an additional analysis comparing matched hospital-based controls (n = 1,438) to cases (n = 719) at a ratio of 2:1 based on age, sex, obesity, hypertension, type 2 diabetes, and coronary artery disease. The major finding of this study was that increased HDL-C or apolipoprotein A1 levels measured many years before the onset of COVID-19 infections was associated with a reduced risk of developing COVID-19 infection. A 10 mg/dl increase in HDL-C or apolipoprotein A1 levels was associated with ∼10% reduced risk of COVID-19 infection. In addition, an increased risk of death from COVID-19 infections was also inversely related to HDL-C and apolipoprotein A1 levels. In some analyses, increased triglyceride levels were also associated with an increased risk of COVID-19 infections. In contrast, increased LDL-C and apolipoprotein B levels were not associated with an increased risk of COVID-19 infections. To determine if this HDL-C protection from COVID-19 infections was the causal link, this study also evaluated the genetic effects of increased HDL-C using a genetic risk score based on SNPs and Mendelian Randomization but did not find an association of increased HDL-C levels and a decreased risk of COVID-19 infections. This failure to demonstrate an association could be due to the relatively small number of individuals in this study compared with the studies of Madsen and Trinder described above, which found a causal relationship between HDL-C and infections but studied a much larger number of individuals. Larger studies or meta-analyses of several smaller studies are needed to more definitively determine if there is a causal link between HDL-C levels and the risk of COVID-19 infections.

Finally, Hilser *et al.* (62) also confirmed prior studies that individuals with homozygosity for apolipoprotein E4 have a 2- to 3-fold increased risk of severe COVID-19 infections (63, 64) and that this was not due to dementia or Alzheimer's disease. Studies have shown that patients who are apolipoprotein E3/4 have an increased inflammatory response to toll receptor ligands compared with patients who are apolipoprotein E3/3, which could result in an increased risk of a more severe response to COVID-19 infections (65). African Americans have an increased frequency of the E4 allele, which could be one factor that contributes to the increased severity of COVID-19 infections in this group (66). In addition, in patients with HIV, apolipoprotein E4/4 is associated with an accelerated disease progression and death compared with apolipoprotein E3/3 (67).

Several other studies using the UK BioBank have also demonstrated that low HDL-C were associated with an increased risk of COVID-19 infections (68, 69, 70, 71). Aung *et al.* additionally reported that LDL-C and triglycerides levels were not associated with COVID-19 infections (69), whereas Scalsky and colleagues observed that elevated apolipoprotein A1 levels were associated with a reduced risk of testing positive for SARS-CoV-2 while LDL-C, apolipoprotein B, and triglyceride levels were not found to be significantly associated with an increased risk (70). However, Zhang *et al.* found that increased triglyceride levels were associated with an increased risk of COVID-19 infection (71). Thus, there is consistent evidence that baseline HDL-C and apolipoprotein A1 levels play a role in determining the risk of developing COVID-19 infections. The effect of baseline triglyceride levels requires additional study.

Two studies have used a genetic approach to determine if lipoproteins play a causal role in COVID-19 infections. Ponsford et al using the UK BioBank (10,154 cases and 452,764 controls) and HUNT Study (Trøndelag Health Study—2,301 cases and 67,121 controls) databases reported that there was no evidence supporting an association of genetically induced LDL-C with the risk for severe COVID-19 infections (72). In contrast, Aung *et al.* also using the UK BioBank (1,211 cases and 387,079 controls) reported that genetically higher exposure to LDL-C was associated with an increased risk of COVID-19 (69). Clearly additional studies are required to determine if there is a causal relationship between LDL-C, HDL-C, or triglycerides with the risk of COVID-19 infections.

The potential HDL-C–mediated protection from COVID-19 could be due to HDL-C levels having beneficial effects on the host’s immune response to infection and/or to HDL-C levels having an inhibitory effect on viral replication. Studies have shown that HDL-C can modulate innate and adaptive immunity that could increase resistance to viral infections (73). Moreover, studies have shown that HDL-C and apolipoprotein A1 have antiviral properties (74, 75, 76). Furthermore, a recent study has shown that HDL has antiviral activity against SARS-CoV-2 (77). In addition, D-4F, an apolipoprotein A-I mimetic peptide, reduced the severity of influenza in an animal model providing further evidence of potential benefits of HDL and apolipoprotein A-I in viral infections (78).
